# A New Remedy

**Published:** 1885-01

**Authors:** 


### A NEW REMEDY.

Years ago *Punch* published a picture representing an errand boy explaining to a horrified doctor that "Missus had taken the lotion and rubbed her leg with the mixture, but felt no better." The picture is forcibly recalled to memory by a newspaper item which records that a woman in this city, for whom a doctor had recently prescribed a porous plaster, mistook the proper use of that article, and administered it to herself internally.

The result of this novel remedy seems to have been on the whole successful. The plaster did not relieve the pain in the chest for which it was originally prescribed, but it strengthened the woman and restored her appetite. It appears that she was living at a cheap boarding house, where fried beefsteak was the chief article of diet, and she found the porous plaster by contrast tender and appetizing. When, on the day after prescribing the plaster, the physician made his usual visit, he found his patient earnest in praise of his palatable and beneficial medicine, and being a liberal minded and enterprising physician, he immediately directed her to take one plaster daily for the next five days, at the expiration of which time she was out of all danger.

In spite of the success of this new remedy, it is doubtful if it will prove beneficial to all patients suffering from affections of the chest and lungs. It is probable that a patient, in order to be benefitted by porous plasters taken internally, must be accustomed to a cheap boarding house table. Other patients would be sure to find these plasters unpalatable, and even indigestible. The doctor who claims to have discovered the new remedy should follow the precedent of the naval surgeon who, having failed to cure a sailor with two legs by a remedy which had once cured a sailor with a wooden leg, decided that the remedy was adapted only to wooden-legged men. Porous plaster is doubtless valuable when taken internally by boarders, but it remains to be seen if it is available in other cases.—*New York Times.*

